# Aberrant brain language network in schizophrenia spectrum disorder: a systematic review of its relation to language signs beyond symptoms

**DOI:** 10.3389/fpsyt.2024.1244694

**Published:** 2024-07-04

**Authors:** María F. Alonso-Sanchez, Lucía Z-Rivera, Mónica Otero, Jorge Portal, Álvaro Cavieres, Pedro Alfaro-Faccio

**Affiliations:** ^1^ Escuela de Fonoaudiología, Centro de Investigación del Desarrollo en Cognición y Lenguaje (CIDCL), Facultad de Medicina, Universidad de Valparaíso, Viña del Mar, Chile; ^2^ Advanced Center for Electrical and Electronic Engineering (AC3E), Universidad Técnica Federico Santa María, Valparaíso, Chile; ^3^ Facultad de Ingeniería, Arquitectura y Diseño, Universidad San Sebastián, Santiago de Chile, Chile; ^4^ Centro BASAL Ciencia & Vida, Universidad San Sebastián, Santiago de Chile, Chile; ^5^ Departamento de Electrónica, Univeridad Técnica Federico Santa María (USM), Valparaíso, Chile; ^6^ Departamento de Psiquiatría, Escuela de Medicina, Universidad de Valparaíso, Valparaíso, Chile; ^7^ Instituto de Literatura y Ciencias del Lenguaje, Pontificia Universidad Católica de Valparaíso, Valparaíso, Chile

**Keywords:** linguistic, brain, fMRI, psychosis, semantics, language models, brain connectivity, brain volume

## Abstract

**Background:**

Language disturbances are a core feature of schizophrenia, often studied as a formal thought disorder. The neurobiology of language in schizophrenia has been addressed within the same framework, that language and thought are equivalents considering symptoms and not signs. This review aims to systematically examine published peer-reviewed studies that employed neuroimaging techniques to investigate aberrant brain-language networks in individuals with schizophrenia in relation to linguistic signs.

**Methods:**

We employed a language model for automatic data extraction. We selected our studies according to the PRISMA recommendations, and we conducted the quality assessment of the selected studies according to the STROBE guidance.

**Results:**

We analyzed the findings from 37 studies, categorizing them based on patient characteristics, brain measures, and language task types. The inferior frontal gyrus (IFG) and superior temporal gyrus (STG) exhibited the most significant differences among these studies and paradigms.

**Conclusions:**

We propose guidelines for future research in this field based on our analysis. It is crucial to investigate larger networks involved in language processing, and language models with brain metrics must be integrated to enhance our understanding of the relationship between language and brain abnormalities in schizophrenia

## 1 Introduction

Language disturbances are a fundamental characteristic of schizophrenia (SZ) (1, 2). These abnormalities have been described as the expression of the formal thought disorder (FTD), whether as part of the positive (speech disorganization) or negative (speech impoverishment) symptoms (3). Researchers have examined the neurobiology of language disturbances in SZ through the framework of FTD (4–10). FTD was conceptualized since Kraepelin’s early work on incoherence descriptions (1) and loosening of associations by Bleuler (4). Since then, it has been associated with various aspects of language and communication. The assessment of FTD involves the use of speech-based scales, such as Andreasen’s (5) Thought, Language, and Communication (TLC) or Liddle’s (6) Thought and Language Index (TLI), which subjectively capture discourse features. It is important to note that these scales include items that cluster into subscales related to impoverished thought/language (such as poverty of speech and weakening of goal) and disorganized thought/language (including looseness, peculiar words, sentences, and logic). Thus, within this framework, language and thought are essentially considered equivalent.

Neuroimaging studies have provided evidence that FTD in SZ is associated with alterations in the brain’s language network (7–10). The relationship between FTD and the brain language network suggests that the impaired organization of thoughts and speech observed in individuals with SZ may stem from underlying neural dysfunctions in language-related brain regions. Specifically, abnormalities have been observed in areas such as the prefrontal cortex (PFC), inferior frontal gyrus (IFG), temporal lobe, and superior temporal gyrus (STG), which are known to play critical roles in language comprehension and production (7, 8). Disruptions in the connectivity and activation patterns within these regions have been associated with the presence and severity of FTD. These dysfunctions can impede the integration and coordination of different linguistic processes, leading to the characteristic disorganized and incoherent speech patterns observed in FTD. In fact, according to Wensing´s (7) review, three clusters show convergent aberrant activation linked to FTD: the left STG was associated with speech and auditory processing, and two clusters in the left posterior middle temporal gyrus (MTG) were involved in semantic processing.

However, it is crucial to acknowledge that different levels of language (i.e. morphology, syntax, lexical) entail distinct cognitive challenges and may involve different brain regions. In the same vein, it is also essential to consider that these language levels do not function autonomously but rather interact with each other and that linguistic meaning is generated as a result of this interconnection. Therefore, it is important not to confuse micro processes, such as the processing of lexical units (lexical meaning), and macro processes, such as the interpretation of a text based on the context in which it occurs (semantic meaning).

We sacrifice specificity when investigating brain activation patterns using macro linguistic markers or complex symptoms such as FTD. For instance, when we analyze the weakening of goal, we are actually analyzing the product of numerous linguistic and cognitive micro-processes working in tandem. Examining language disorders and their connection to the brain in detail, focusing on the signs of linguistic micro processes, can provide us with more precise insights. For instance, in the domain of semantics, it is important to differentiate between lexical access and sentence comprehension despite their shared involvement in meaning. By delving into these granular aspects, we can gain a deeper understanding of the complexities of language atypicalities and their neural mechanisms.

In the context of exploring language networks in individuals with SZ, it is especially important to focus on signs rather than symptoms. While symptoms provide valuable insights into the patient’s personal experience, signs provide specific measures of the language disorder. Previous reviews have focused on subjective observation-based instruments to measure symptoms of thought/language disturbances in SZ. While these rating scales have proven clinically useful, they may not fully capture subtle deviations across all dimensions of language. Alternatively, tools from cognitive neuroscience, neuropsychology, and computational sciences offer a better opportunity to explore the multidimensional nature of language. Experimental paradigms such as lexical decision, semantic integration, and semantic association provide insights into cognitive subprocesses involved in language processing. Additionally, computational linguistic tools enable quantitative analyses such as part-of-speech tagging, syntactic parsing, semantic similarity, and speech graphs, among other approaches, to provide objective patterns and specific measures of language impairments. By leveraging language models, linguistic features extracted from oral or written text can be employed to explore the relationship between language impairments and the underlying neural networks in SZ.

Understanding the relationship between language and language networks in patients with SZ is essential for improving diagnostic tools and developing targeted interventions. By combining cognitive neuroscience, linguistic and computational linguistic techniques with neuroimaging data, we can enhance our understanding of the language network functioning in persons with SZ, improve diagnostic approaches, and advance the development of targeted interventions of neuromodulation for language impairments in this population. In this context, the aim of this review is to analyze the brain language networks and related areas in individuals with Psychosis spectrum in relation to linguistic signs.

## 2 Materials and methods

### 2.1 Eligibility criteria

#### 2.1.1 Population

We include in this review all the studies with first episode of psychosis, high-risk and chronic schizophrenia patients regardless of their age, sex, educational level, ethnicity or language. We exclude all the studies that did not include a structural (MRI), hemodynamic [functional Magnetic Resonance Imaging (fMRI) or functional Near-Infrared Spectroscopy (fNIRS)] and a language measure (cognitive paradigms, neuropsychological or computational linguistic metrics).

These criteria were chosen to sift through the vast array of information concerning schizophrenia and were bolstered by a systematic search strategy, explained below. Our review aimed to analyze the intersection of language, neuroimaging, and schizophrenia.

#### 2.1.2 Interventions

Not applicable since the review addresses the relationship between linguistic and brain measures in SZ patients.

#### 2.1.3 Comparators

We included comparisons with healthy controls (HC) groups and other psychiatry pathologies [eg. depression or bipolar disorder (BD)]. However, it is worth noting that depending on the methodology employed, certain studies did not utilize a separate HC group, opting instead for a control task or paradigm within the same group. For example, some studies included patients with schizophrenia with and without hallucinations. We have included these studies in our analysis to encompass a broader range of research approaches.

#### 2.1.4 Outcomes

In this review, we defined outcome measures of brain structure or function and language described as follows. Brain structure: whole brain volume, white matter tracts, grey matter volume. Brain function: bold signal contrast, functional connectivity, cortical gradient and effective connectivity. Language: discourse analysis including natural language processing tools (eg. part of speech tagging, syntactic complexity, semantic similarity), lexical decision paradigms, semantic comprehension and judgment paradigms, multimodal integration (eg. gestual), verbal fluency and semantic strategies for memory encoding.

### 2.2 Sources and search strategy

The objective of this study was to conduct a systematic review of peer-reviewed, published studies that utilized neuroimaging to investigate the abnormal brain language networks in individuals with SZ. To achieve this, a comprehensive search was performed using the search strategy described in the section below, and relevant information regarding the study design, experimental design, and other details were extracted (refer to **Supplementary Material Worksheet** named **“Data extracted.csv”**).

#### 2.2.1 Search strategy

**Figure 1:**
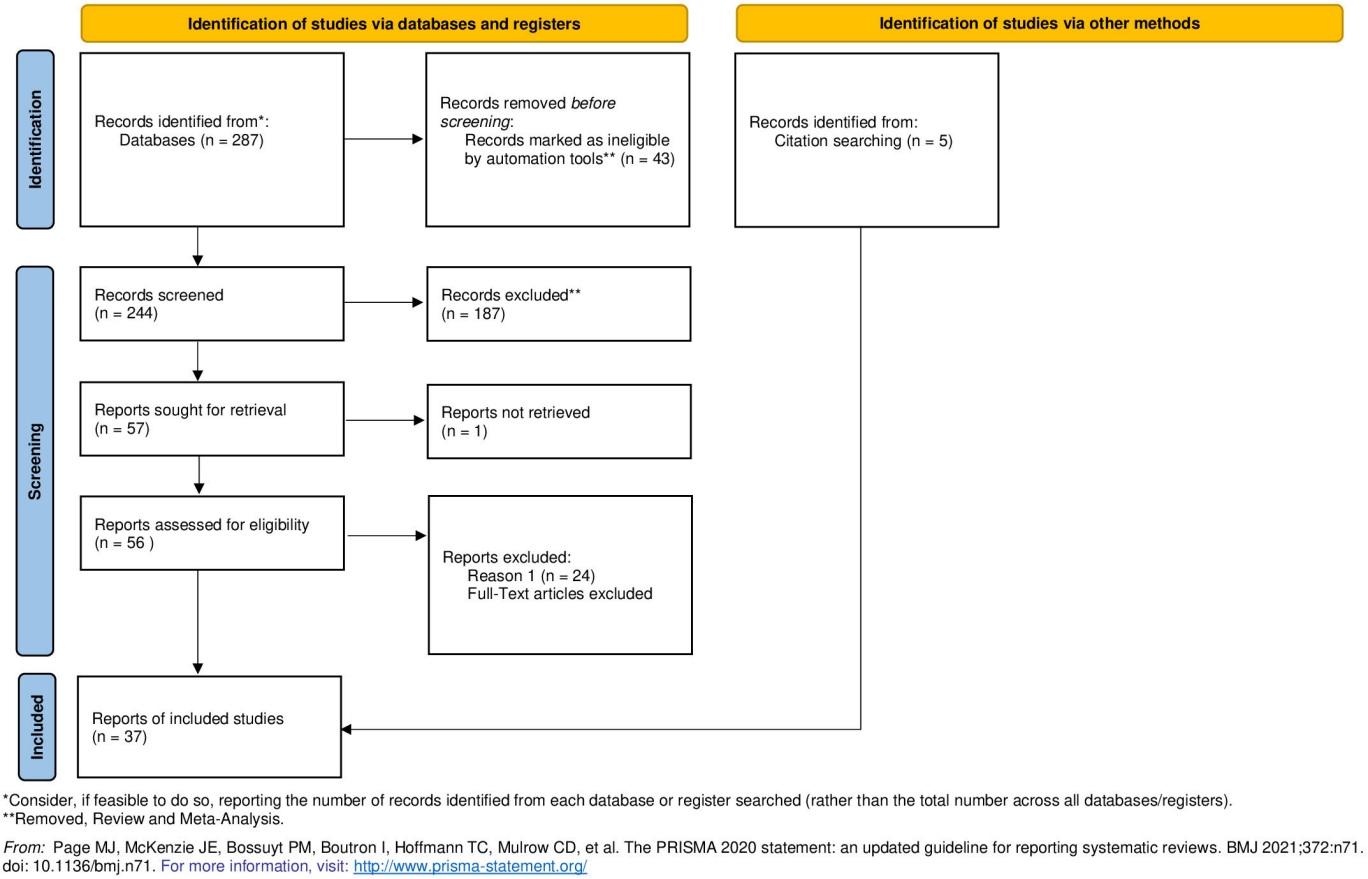
Primas flowchart.

A search for articles was conducted in the PubMed database until January 2024. We employed the Entrez interface to extract information efficiently from the database (11, 12). This approach leverages the logical relationships between individual entities in the database, resembling relational natural language (13). To execute queries on the database, we utilized the BioPython library integrated with Entrez (14). Initially, access to the MEDLINE/PubMed search engine API (Application Programming Interface) must be requested, and upon approval, an API key is assigned to the user for query execution. APIs serve as software components, enabling developers to effortlessly integrate search functionalities into websites and applications. The search logic is based on keywords or the presence of specific words in the text, and logical connectors such as OR and AND form the QUERY, as follows:

The search strategy consisted of three domains: (I) Language-related terms, (II) Brain Neuroimaging terms, and (III) Schizophrenia-related terms. These domains were combined using the logical operator “AND” between the domains and “OR” within domain keywords. Hence, the search included the following boolean keywords: (speech OR discourse OR semantic OR lexical OR grammar OR syntactic OR coherence OR syntax OR nouns OR verbs) AND (connectivity OR cortical gradient OR effective connectivity OR functional connectivity OR cortical volume OR Broca OR perisylvian OR Wernicke OR white matter tracts OR grey matter) AND (Psychosis OR Schizophrenia Spectrum). PubMed’s search strategy encompassed the [‘All Fields’] and [‘Mesh Term’] sections. Our article search and selection process is shown in **Figure 1**. The initial search yielded 287 research articles without any duplicates. Review papers and meta-analyses (a total of 43) were excluded from the papers to be summarized in this review. However, some were referenced and used for discussion or as additional sources of information. Consequently, a total of 244 papers remained for information extraction. Seven additional papers were identified from other sources and added to the papers screening. After identifying these items we analyzed the cause behind their absence in the automated search. Our analysis revealed that the mesh terms were unavailable, excluding these items from the initial search. For all 249 papers, full-text documents, including abstracts, methods, results, and conclusions sections, were extracted. Articles lacking a discussion section were excluded from further analysis. Only one full-text document was not obtained as it was not available online (15). Then, using the abstracts of these studies, we excluded those that only partially addressed the language measures or the neuroimaging criteria or that did not involve ultra-high risk (UHR), first-episode, or chronic SZ patients, resulting in the exclusion of 187 articles. After this process, we obtained 62 articles for further analysis. From these studies, some were further excluded due to the absence of linguistic or neuroimaging data, or the analysis of the relationship between these variables. As a result, we retained a total of 39 articles (**Figure 1**).

#### 2.2.2 Data extraction

After the article search, some automatic data extraction was performed using the BertForQuestionAnswering tool, a specific model architecture based on the Bidirectional Encoder Representations from Transformers (BERT) (16) framework, designed specifically for question-answering tasks. The model was trained using the Stanford Question Answering Dataset (SQuAD) (17) and re-trained using the specific dataset extracted from the PubMed database to enable this step. The limitation of the SQuAD dataset lies in its reliance on general knowledge derived from a set of Wikipedia articles. In our approach, we addressed this limitation by enhancing the dataset through the incorporation of PubmedQA, thereby adding a specialized dimension to the knowledge base. Previous research has shown the efficacy of pre-trained BERT models in developing dedicated question-and-answer systems (18, 19). The data extracted through this process is comprehensively presented in the **Supplementary Material** **Table ‘Data extracted.csv.’** This automated extraction method significantly improved the retrieval of information from the selected articles. Nevertheless, to ensure accuracy and reliability, the resulting table underwent a meticulous manual review and correction process conducted by the authors of the paper.

The questions generated to populate this table are as follows:

1. To extract sample size: What is the sample size of the study? or How many patients were included in the experiment? or How many subjects were involved in the study?
2. To extract the gender composition: What was the gender composition of the patient group? or What is the distribution of the study group’s sex?
3. To extract information about the distribution of ages: What are the ages of the participants?
4. To extract information about the inclusion criteria of the studies: What is the study’s inclusion criteria?

### 2.3 Study selection, quality assessment and risk of bias

For the study selection, we follow the Preferred Reporting Items for Systematic Reviews and Meta-Analyses (PRISMA) (20) recommendations: identification, screening, eligibility and inclusion. The criteria for selection were to address the language brain network using linguistic measures and brain measures. Given our emphasis on brain mapping, we opted for hemodynamic measurements as they offer superior spatial resolution compared to other methods. By utilizing these measurements, we can obtain detailed information about brain activity and map out specific regions more accurately and precisely. A third-party consensus resolved disagreements on data selection. Then, with the selected articles, the quality assessment of each article was done by two independent authors with the Strengthening the Reporting of Observational Studies in Epidemiology (STROBE) (21) guidance analyzing the following parameters: settings of data collection, participant eligibility criteria, defined and quantified variables, sources of data and detailed measurements, efforts to address potential sources of bias, explanation of how the study size was determined and reported the statistical analysis. Based on our quality assessment, all the selected articles were deemed suitable for inclusion in this review (**Table 1**).

**Table 1:** Quality assessment of the studies.

Author	year	Settings	Participants	Variables	Data source	Bias	Study size	Quantitative variables	Statistics Methods
Alonso-Sánchez et al. (22)	2022	✔	✔	✔	✔	✔	X	✔	✔
Roes et al. (23)	2023	✔	✔	✔	✔	✔	X	✔	✔
Adamczyk et al. (24)	2021	✔	✔	✔	✔	X	✔	✔	✔
Haas et al. (25)	2020	✔	✔	✔	✔	✔	✔	✔	✔
Surbeck et al. (26)	2020	✔	✔	✔	✔	✔	✔	✔	✔
Wroblewski et al. (27)	2020	✔	✔	✔	✔	X	✔	✔	✔
Vanes et al. (28)	2019	✔	✔	✔	✔	X	✔	✔	✔
Vandevelde et al. (29)	2018	✔	✔	✔	✔	✔	✔	✔	✔
Zhang et al. (30)	2016	✔	✔	✔	✔	✔	✔	✔	✔
Iwashiro et al. (31)	2016	✔	✔	✔	✔	✔	X	✔	✔
Holper et al. (32)	2015	✔	✔	✔	✔	X	✔	✔	✔
Woodward et al. (33)	2015	✔	✔	✔	✔	✔	✔	✔	✔
Royer et al. (34)	2015	✔	✔	✔	✔	✔	✔	✔	✔
Hatton et al. (35)	2014	✔	✔	✔	✔	✔	✔	✔	✔
Lavigne et al. (36)	2015	✔	✔	✔	✔	✔	✔	✔	✔
Rannikko et al. (37)	2012	✔	✔	✔	✔	✔	✔	✔	✔
Allen et al. (38)	2012	✔	✔	✔	✔	✔	X	✔	✔
Meijer et al. (39)	2011	✔	✔	✔	✔	✔	✔	✔	✔
Van Veelen et al. (40)	2011	✔	✔	✔	✔	✔	X	✔	✔
Dickey et al. (41)	2010	✔	✔	✔	✔	✔	✔	✔	✔
Bhojraj et al. (42)	2009	✔	✔	✔	✔	✔	X	✔	✔
Bleich-Cohen et al. (43)	2009	✔	✔	✔	✔	✔	X	✔	✔
Habets et al. (44)	2008	✔	✔	✔	✔	✔	X	✔	✔
McIntosh et al. (45)	2008	✔	✔	✔	✔	✔	✔	✔	✔
Sabb et al. (46)	2010	✔	✔	✔	✔	✔	✔	✔	✔
Chen et al. (47)	2013	✔	✔	✔	✔	X	X	✔	✔
Han et al. (48)	2007	✔	✔	✔	✔	✔	✔	✔	✔
Horn et al. (49)	2012	✔	✔	✔	✔	✔	X	✔	✔
Kircher et al. (50)	2005	✔	✔	✔	✔	✔	X	✔	✔
Kircher et al. (51)	2002	✔	✔	✔	✔	✔	X	✔	✔
Kircher et al. (52)	2008	✔	✔	✔	✔	✔	X	✔	✔
Kubicki et al. (53)	2003	✔	✔	✔	✔	X	✔	✔	✔
Kuperberg et al. (3)	2007	✔	✔	✔	✔	X	✔	✔	✔
Kuperberg et al. (54)	2019	✔	✔	✔	✔	X	✔	✔	✔
Ragland et al. (55)	2008	✔	✔	✔	✔	✔	X	✔	✔
(Sass et al. (56)	2014	✔	✔	✔	✔	✔	✔	✔	✔
Tagamets et al. (57)	2014	✔	✔	✔	✔	✔	X	✔	✔
Wilson et al. (58)	2013	✔	✔	✔	✔	✔	✔	✔	✔
Liang et al. (59)	2022	✔	✔	✔	✔	✔	✔	✔	✔

✔: reported by the authors. X: not reported by the authors.

### 2.4 Data synthesis

**Figure 2:**
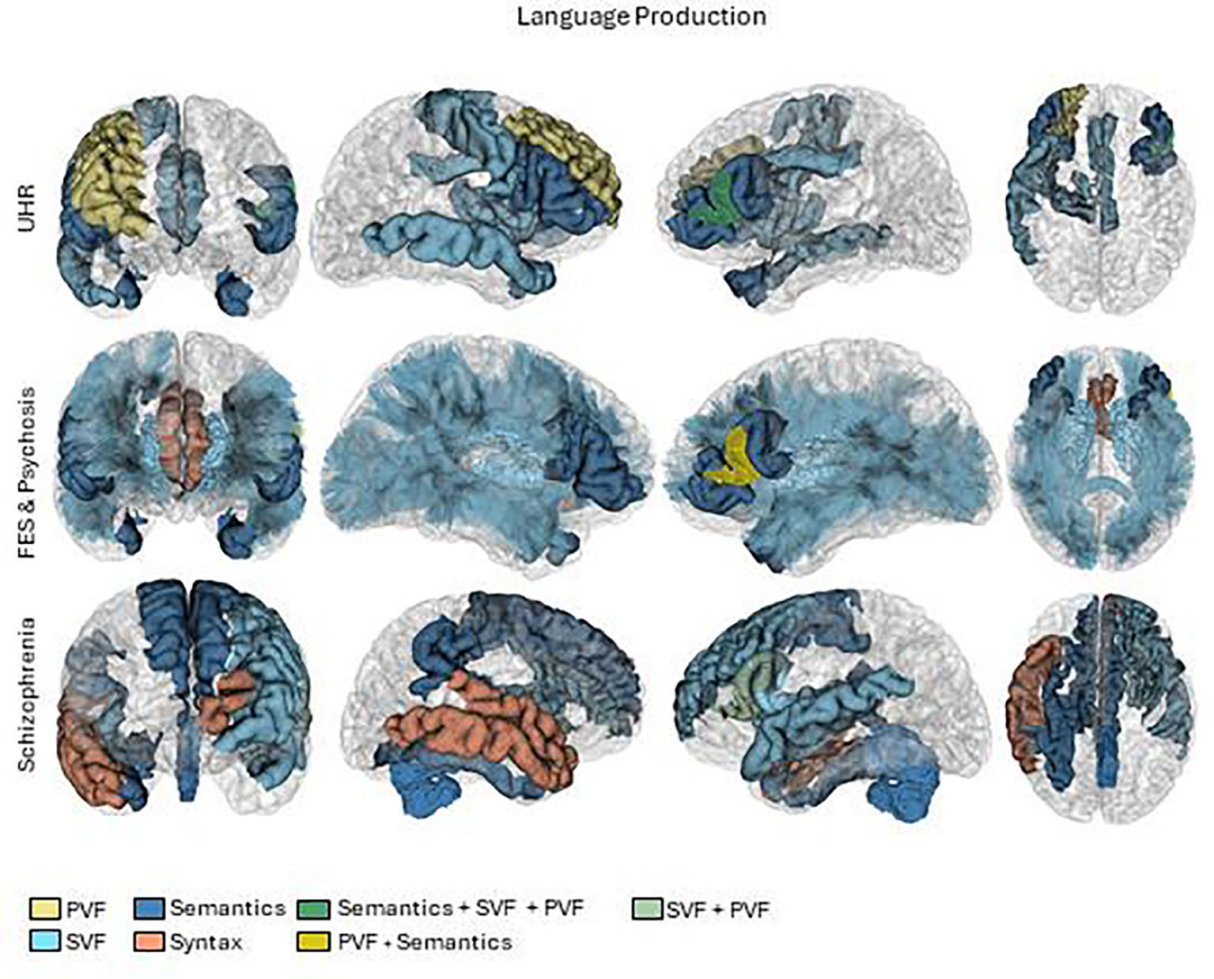
Brain activations in language production tasks. First row UHR (Ultra high risk), second row FES (First episode schizophrenia) and psychosis and third row Schizophrenia. PVF, Phonological verbal fluency.

**Figure 3:**
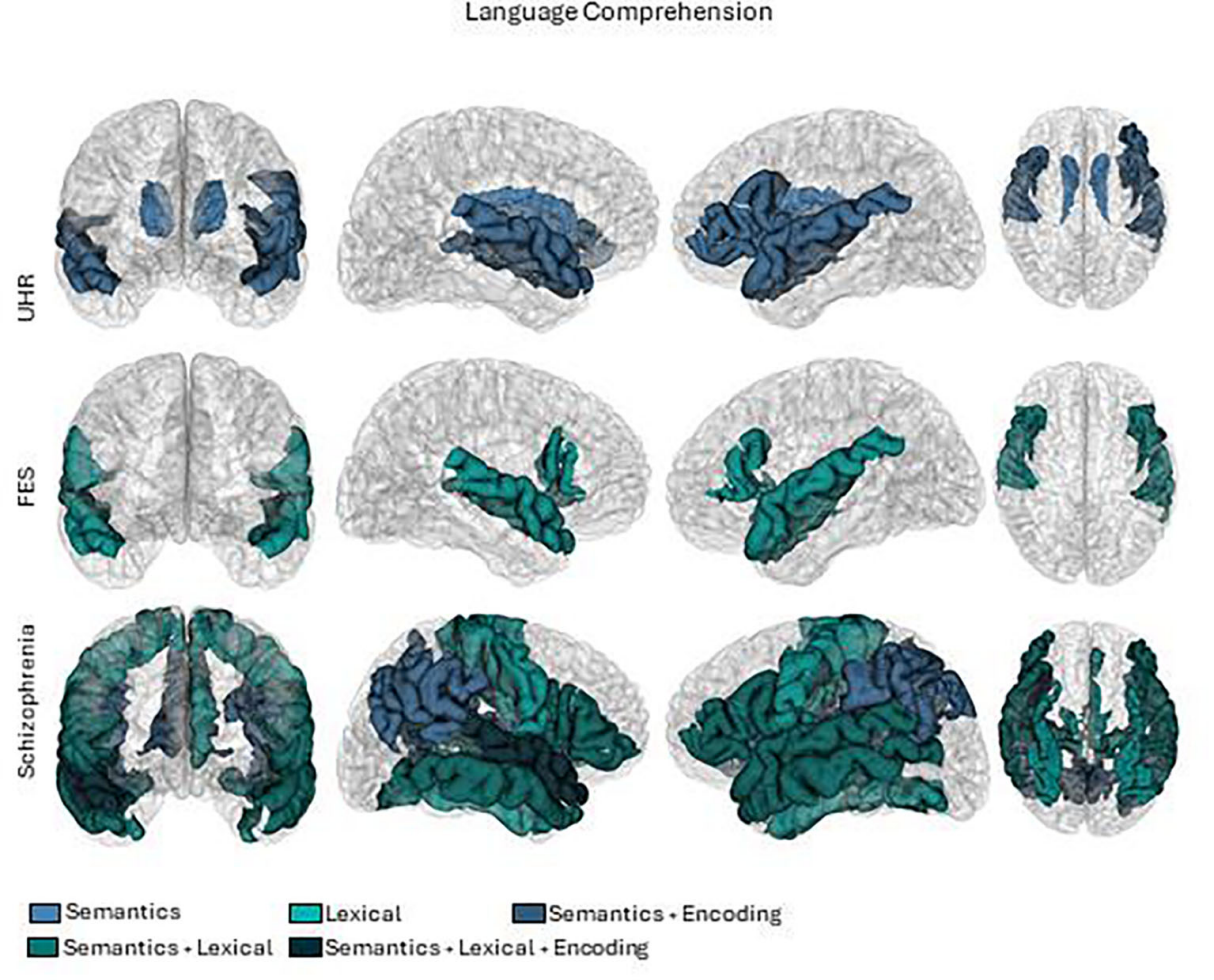
Brain activations in language comprehension tasks. First row UHR (Ultra high risk), second row FES (First episode schizophrenia) and third row Schizophrenia.

We critically appraised each study to compile a synthesis. We summarized the results considering participants, language and brain measures, and principal outcomes. To outline the results, we grouped the studies according to the type of patients (High risk, first episode, and chronic) and the type of language measure (including semantic encoding memory, verbal fluency, lexical, syntactic, or semantic). To generate the brain summary figures (**Figures 2**, **3**), we used pre-processed data from one subject of the HCP database (60). Probabilistic tractography was computed from diffusion MRI data using MRtrix3 software (61). The TractSeg algorithm (62) was then applied to automatically segment white matter bundles. Brain parcellations, represented by different colors in the figures, were used to identify regions of interest (63–65).

## 3 Results

As mentioned above, we analyzed the results of 39 studies based on the type of patients, type of language task and brain measure. In the following sections, we discuss the findings of this review based on the production and comprehension of language.

### 3.1 Production of language

In the production dimension of language, we categorized the results by verbal fluency, syntax and semantics. The results are summarized in **Tables 2**–**4**, and the patterns of brain activations are shown in **Figure 2**.

**Table 2:** Summary of relevant findings in verbal fluency.

Study	Participants	Linguistic measure	Brain Measure	Results
Holper et al 2015 (32)	PA: 66 PS:39 PA-PS:55 HC:28	Verbal fluency (phonemic and semantic)	fNIRS	subthreshold psychotic symptoms individuals showed reduced hemodynamic responses in VF, as well as weaker connectivity between prefrontal and temporal cortices compared to controls.
Meijer et al. 2010	UHR:37	Verbal fluency (semantic)	MRI	In UHR, lower SVF scores were correlated with decreased grey matter density in the right STG and MTG, right insula, and left ACC
Bhojraj et al., 2009 (42)	UHR:60 HC:42	Verbal fluency	MRI	UHR group display deficits in VF reductions in gray matter volume and reversed asymmetry of the pars triangularis.
Allen et al. 2012 (38)	UHR: 41 HC:24	Verbal fluency (semantic)	fMRI-PET	UHR showed increased activation in bilateral PFC, brainstem (midbrain/basilar pons), left hippocampus, and greater midbrain-PFC connectivity.
Iwashiro et al 2016 (31)	UHR: 23 FES:18 HC:16	Verbal fluency (phonological)	fNIRS-MRI (GMV)	The UHR and FES groups exhibited reduced brain activity in the left pars triangularis compared to the control group.
Vanes et al., 2019 (28)	FES: 87 HC:35	Verbal fluency (phonological and semantic)	MRI (MWF)	Psychosis patients exhibited decreased myelin content in the left SLF, SLFT, ILF, which fully mediated VF task performance.
Habets et al 2008 (44)	Psychosis:31 Non-psychotic:32 HC:28	Semantic memory-encoding - Verbal fluency	MRI	VF and grey matter density were negatively correlated in the basal ganglia and positively correlated in the thalamus.
Hatton et al 2014 (35)	Psychosis:42 HC:45	Verbal fluency (semantic)	MRI	Patients with psychosis exhibited fractional anisotropy reductions in left SLF, SLFT, ILF and forceps major, which were associated with impaired SVF
Kircher et al 2008 (52)	SZ: 12 HC:12	Semantic judgments - Verbal fluency (phonological and semantic)	fMRI (BOLD contrast)	In SZ, there was a reduction in left hippocampal activity during semantic tasks and a failure to engage the anterior cingulate gyrus during VF tasks.
Ragland et al 2007	SZ: 13 HC:14	Verbal fluency with switching conditions	fMRI (BOLD contrast)	SZ showed increased activation in left PFC, right ACC, right STGl, bilateral thalamus, and left parietal regions during demanding semantic category retrieval
Vandevelde et al 2017	SZ:15 HC:20 BD:14	Verbal fluency - mentally generated verbs	fMRI (FC-SC)	SZ demonstrated reduced functional connectivity in medio-frontal cortex – left subcortical regions compared to BD and left frontolateral compared to HC

**Table 3:** Summary of relevant findings in syntax-production.

Study	Participants	Linguistic measure	Brain Measure	Results
Kircher et al 2005 (50)	SZ:6 HC:6	Speech samples	fMRI (BOLD contrast)	In SZ, there was no correlation observed between the production of complex sentences and activation in the posterior portion of the right MTG and left SFG
Kircher et al 2002 (51)	SZ:6 HC:6	Speech samples	fMRI (BOLD contrast)	In SZ, the amount of speech produced was predominantly correlated with activation in right STG. Conversely, in HC the correlations were found in the left STG.
Liang et al 2022 (59)	FES:66 HC:36	Speech samples	MRI	In FES the widespread cortical thinning was related to reduced syntactic complexity.

**Table 4:** Summary of relevant findings in semantic-production.

Study	Participants	Linguistic measure	Brain Measure	Results
Haas, et al. 2020 (25)	CHR:46 HC:22	Speech samples	rs-fMRI (FC-SC)	There was no difference between groups in pattern of covariation between linguistic features and brain morphometry and RSN connectivity.
Tagamets et al 2014 (57)	SZ:11 HC:11	Speech samples	fMRI (BOLD contrast)	In SZ, coherence was mainly associated with auditory and visual regions depending on the monitoring modality, but STC and MTC showed coherence regardless of task.
Alonso-Sánchez et. al. 2022	FES:30 HC:30	Speech samples	fMRI (EC)	Semantic similarity was associated with higher self-inhibition on IFG and vATL. There was no difference between groups.

#### 3.1.1 Verbal fluency

Out of the nine verbal fluency studies included, one was conducted on subthreshold psychotic symptoms individuals, four of them focused on participants at UHR, two on patients with first-episode schizophrenia (FES) and four on Chronic patients (SZ). Two of the UHR studies utilized structural brain measures (39, 42), while the other two employed functional brain measures (62, 63). In the following section, we review the main findings by group (UHR, FES or CHR) and by type of brain measure (structural or functional), as shown in **Table 2**.

Holper’s (32) study utilized fNIRS on 188 subjects categorized into four groups based on varying levels of subthreshold psychotic symptoms: HC group (n=28), Paranoia group (n=66), Psychoticism group (n=39), and Paranoia-Psychoticism group (n=55). The findings revealed diminished hemodynamic responses during phonological and semantic tasks in the prefrontal and temporal cortex among individuals with high subthreshold psychotic symptoms compared to the HC group (32).

Regarding the population with ultrahigh risk and structural measures, some studies observed a significant positive correlation between semantic verbal fluency scores and grey matter density in specific brain regions (39, 44). Meijer (n=37) observed this correlation in the right STG and MTG (BA 21), right insula (BA 13), and left anterior cingulate cortex (BA 31 and 32) (39) while Habets (n=32) found it primarily in the basal ganglia, nucleus caudatus, striatum, and insula (44). Additionally, a similar correlation was observed but involving a phonological instead of semantic task, characterized by reductions in gray brain matter volume and a reversed asymmetry, particularly in the pars triangularis (BA 45) (42). In examining the functional activity of the brain during phonological verbal fluency tasks in the same population, compared to controls, some authors found increased activation in the right middle and superior frontal gyri, with a heightened response in the dorsolateral prefrontal cortex (BA 9 and 10) (38). Conversely, another study observed decreased brain activity in the left pars triangularis (BA 45) using fNIRS (31). These brain areas are strongly associated with executive and language-related tasks. Allen (38) further investigated participants who transitioned to psychosis versus those who did not, noting greater activation in the right inferior frontal, bilateral middle frontal (BA 10), and left superior frontal gyri in the transition group, along with increased midbrain-prefrontal cortex functional connectivity (38).

In FES patients (n=87) Vanes (28) observed reduced myelin water fraction (MWF) in the left temporal white matter compared to HC. Moreover, in patients, MWF in the left inferior fronto-occipital fasciculus (IFOF) and inferior longitudinal fasciculus (ILF) demonstrated a positive correlation with intelligence quotient and verbal fluency. These factors fully mediated the group differences in performance for both PVF and SVF tasks (28).

Involving patients with psychosis we found two studies, one analyzed grey matter density while the other focused on fractional anisotropy and axial diffusivity. In Habets’ study (44), the patient group exhibited a significant negative correlation with grey matter density in specific regions of the basal ganglia, notably the nucleus caudatus, putamen, and globus pallidum, alongside a positive correlation with grey matter density in the caudate nucleus and the thalamus. Additionally, in the relatives group, a positive correlation was observed between verbal fluency and grey matter density in the basal ganglia, nucleus caudatus, striatum, and insula (44). Conversely regarding the white matter, Hatton’s study (35) found that the psychosis group (n:42), in comparison to the HC group (n:45), demonstrated diminished fractional anisotropy and axial diffusivity in the left SLF, SLFT, ILF, and forceps major, which was associated with poorer semantic fluency (35).

In patients with schizophrenia, the PVF task revealed significant left-sided inferior frontal activity, with the peak activity observed in left IFG (Brodmann areas 45 and 44). This activity was notably higher when compared to the control condition of repeating the word “pause.” The cluster extended ventrally into Brodmann area 47 and dorsally into adjacent middle frontal areas (Brodmann area 6). Conversely, semantic fluency scores resulted in sparse activity in the left IFG (Brodmann area 44), with some extension into adjacent middle frontal areas (Brodmann area 9) compared to reading (52). While some of these findings align with previous studies, others diverge. These differences may stem from the varying cognitive demands of repeating the word “pause” and reading as control conditions. Ragland´s study (55) addressed the impact of cognitive demand of semantic retrieval on brain activations in individuals with schizophrenia. They used a modified version of the effortful overlearned sequences (e.g., days of the week) versus a single semantic category (e.g., vegetables) paradigm. They included a switching condition to the effortful overlearned sequences (e.g., one, Monday, two, Tuesday) and to the single semantic category (e.g., chair, peach, table, apple). The study included 13 patients with schizophrenia and 14 HC participants. The SZ patients were able to successfully perform semantic word generation tasks even when faced with increased word retrieval and switching demands. On the brain patterns, it was observed that patients exhibited normal activation in the frontal and parietal lobes when generating words associated with familiar sequences, where the demands for retrieval were low. However, when generating words under more challenging conditions (switching categories), patients with schizophrenia showed heightened activation in the left STG compared to the HC group. STG over-activation extended to PFC, middle temporal, parietal, and basal ganglia regions. This suggests that increased cognitive effort is required for individuals with SZ to perform tasks involving semantic word generation in demanding tasks (55). Finally, using a seed analysis during an experimental paradigm of mentally generating verbs in a block design, Vandevelde (15: SZ, 14: BD, and 20: HC) observed a significant reductions in functional connectivity among a left fronto-lateral cluster (including Broca’s area, the left middle frontal gyrus, the prefrontal gyrus and the insula). However, the primary finding indicated that individuals with schizophrenia, in contrast to those with bipolar disorder and healthy controls, exhibited reduced functional connectivity within a specific paired-seed region: medio-frontal cortex (including the left paracingulate gyrus, the superior frontal gyrus and the supplementary motor area) and left subcortical regions (left basal ganglia including the left putamen belonging to the striatum, the left pallidum and the thalamus). This outcome supports the proposition that individuals with schizophrenia experience a distinct reduction in functional connectivity between the medial prefrontal cortex and subcortical regions (29).

In summary, the results indicate that both grey and white matter within the language network are associated with verbal fluency performance. When it comes to functional outcomes, variations arise depending on the task. Some studies focused solely on phonological or semantic verbal fluency, while others introduced variations such as verb mental generation or control conditions like repeating a word or reading. Across all verbal fluency tasks, increased activity in left BA44 and BA45 was consistently observed. However, in one study, this activity was lower but this was compared to a reading task. Moreover, heightened cognitive demands revealed broader area involvement, including MTG, STG, and PFC.

#### 3.1.2 Syntax

Regarding syntax we found three studies and two were from the same authors shown in **Table 3**. Kircher (50, 51) conducted two studies examining the relationship between syntactic production and brain activity. Each study involved six patients with SZ and six HC participants. The participants were asked to provide a three-minute speech sample of picture descriptions, allowing for analysis of the proportion of syntactically simple and complex sentences. In the first study, it was observed that the amount of speech produced among the HC group was primarily correlated with activation in the left STG. In contrast, the patient group exhibited main correlations in the right STG. In the second study, they observed in the HC group that the number of complex sentences produced was correlated with activation in the posterior portion of the right MTG (Brodmann area 21) and the left SFG (BA10). However, no such correlation was observed in the patient group (50, 51). Another research investigation involving 66 patients experiencing first-episode psychosis and 36 healthy controls conducted a comparison of cortical thickness. The results revealed two distinct neuroanatomical subtypes among the patient group. One subtype exhibited near-normal cortical thickness patterns, while the other displayed widespread cortical thinning. In comparison to the subgroup of patients with relatively normal cortical thickness, the subgroup characterized by widespread cortical thinning was older, exhibited higher glutamate concentration in the dACC, and demonstrated reduced mean length of T-units (complexity) in their speech. Additionally, they had lower repeats of content words (lexical cohesion) in their speech, despite maintaining equal fluency in terms of the number of words produced (59). Due to the inherent disparity in brain measurement methodologies, a direct comparison of the results is unfeasible.

#### 3.1.3 Semantics

In the field of semantics, we find 3 studies that analyzed speech samples using computational tools and compared them with neuroimaging measures, as shown in **Table 4**. Haas (25) study (CHR: 46 and HC:22), measured syntactic complexity and semantic coherence with Part of Speech tagging, and Latent Semantic Analysis (LSA) in a description of the life changes they had experienced, the impact of these changes, what had been helpful or unhelpful for them, and their future expectations. The language metrics were used as a covariate of the resting state neuroimaging model. Notably, the results indicated that the observed patterns of covariation were not influenced by the diagnostic status of the participants, suggesting no diagnostic effect on these linguistic patterns. However, the most heavily weighted linguistic features related to functional connectivity were maximum semantic coherence followed by syntactic complexity. Notably, the top-weighted regions mapped near the temporal and prefrontal semantic hubs. Moreover, the brain morphometry was also primarily related to mean semantic coherence, follow by use of interjections and subordinating conjunctions (25). In the same vein, using LSA on interview focused on the topic of “religious belief and computing the cosine angle between pairs of vectors containing the words “myself” and “ourselves), a study (SZ:11 and HC:11) examined the relationship between semantic coherence scores obtained from the discourse and brain activations. The participants underwent an fMRI scanner while performing a word monitoring task. The monitoring modality was manipulated by varying the congruence of auditory and visual stimuli. Between-group analysis was directly performed to compare the correlation between the semantic coherence scores and brain activations in each of the three conditions. The results indicated significant activations in the SZ group compared to the HC group specifically in the homographs condition. Five distinct clusters were identified: the bilateral SFG/supplemental motor area, right supramarginal gyrus, right parahippocampal/FG, right superior temporal pole, and right culmen/vermis (57). Also using resting-state fMRI scans, Alonso et al. (22) conducted a study including 30 individuals experiencing their FES and 30 HC. The study also incorporated semantic distance measures of speech samples. They focused on analyzing the effective connectivity between two crucial nodes of the word production system: the IFG and the ventral anterior temporal lobe (vATL), the semantic hub. The findings revealed a correlation between lexical impoverishment and increased self-inhibition in both the IFG and vATL regions. This heightened self-inhibition was suggested to be associated with a reduction in synaptic gain. Consequently, individuals may be compelled to rely on words that are already activated within the lexical network due to compromised neural activity (22).

The findings from these studies displayed variability due to the diverse cognitive demands associated with the tasks. While two studies utilized resting-state brain measures, the third incorporated a different task with distinct cognitive requirements and brain activations. Unfortunately, none of the studies employed a direct language production task. Nevertheless, when compared to semantic metrics, resting-state measures emerged as superb alternative. Given the rapid nature of word production, the temporal resolution of fMRI may fail to capture much of the variability, and still introduce noise into the signal. Furthermore, during resting state subjects engage in rich mental experiences with a succession of cognitive, emotional and perceptual processes (ref), indeed, semantic activity persists during rest with mind wander, despite instructions to avoid specific task cognitive load (i.e. such as thinking on a pink elephant).

### 3.2 Comprehension of language

In the comprehension dimension of language, since they were all related to semantics, we categorized them by lexico-semantics, semantic meaning, and semantic memory encoding. The results are summarized in **Tables 5**–**7**, and the patterns of brain activations are shown in **Figure 3**.

**Table 5:** Summary of relevant findings in Lexico semantic- comprehension.

Study	Participants	Linguistic measure	Brain Measure	Results
Bleich-Cohen et al 2009 (43)	FES:12 HC:17	Lexical association	fMRI (BOLD contrast)	SZ exhibited reduced lateralization indices in language-related regions, specifically in IFG and STS, as well as increased activation in temporal regions
van Veelen et al 2011 (40)	FES:35 HC:43	Semantic judgments - Lexical association?	fMRI	FES exhibited a significant decrease in the lateralization index, particularly in the IFG and STG.
Han et al 2007 (48)	SZ: 12 HC:12	Lexical association	fMRI (BOLD contrast)	CSZ exhibit aberrant activation in the left IFG and STG regions, which varies based on the degree of word connectivity.
Kuperberg et al 2019 (54)	SZ: 16 HC: 20	Lexical association	fMRI - MEG	SZ exhibit decrease in neural activity in fusiform gyrus
Chen et al 2013 (58)	SZ:20 HC:20	Semantic judgments	fMRI (BOLD contrast)	SZ showed increased left IFG activation and reduced left caudate nucleus activation for meaning-related pairs. The EC indicated weaker caudate nucleus-to-IFG modulation.
Sass et al 2013	SZ: 14 HC:14	Lexical association	fMRI (BOLD contrast)	Semantic relation elicited increased activation in the right AG and precuneus, while modality resulted in decreased activation in the left SFG, MTG, IOG, right AG, and ACC.
Kuperberg et al 2007 (56)	SZ: 17 HC:15	Lexical association	fMRI (BOLD contrast)	CSZ and HC showed different hemodynamic modulation patterns for directly related (inf prefrontal) and indirectly related word pairs (temporal cortices).
Wilson et al 2013 (66)	SZ:17 HC:15	Lexical association	fMRI (BOLD contrast)	HC exhibited hemodynamic suppression in priming. HZ exhibited hemodynamic enhancement in the left fusiform and STG for indirectly related word vs unrelated.
Lavigne et al 2015 (36)	SZ:23 BD:22 HC:27	Lexical association	fMRI (FC)	In hallucinating SZ, a left-dominant temporal-frontal network (left IFG, bilateral STG, FG and SMA) exhibited hypercoupling compared to non-hallucinating patients during speech perception.

**Table 6:** Summary of relevant findings in semantic meaning- comprehension.

Study	Participants	Linguistic measure	Brain Measure	Results
Kubicki 2003 (53)	SZ: 9 HC:9	Semantic judgments	fMRI (BOLD contrast)	SZ exhibited distinct activation patterns for semantically encoded words, including underactivation in the left IFC and overactivation in the left STG.
Royer et al 2015 (34)	SZ:31 BD:20	Speech-listening paradigm	fMRI (FC) (GVAI)	SZ displayed a reduction in leftward functional hemispheric lateralization for language.
Horn et al 2012 (49)	SZ:16 HC:18	Passive word reading paradigm	fMRI (BOLD contrast)	The activation within the semantic network was detected in both groups. In SZ, the severity of FTD was linked to an impairment in the left semantic network.
Kircher et al 2008 (52)	SZ: 12 HC:12	Semantic judgments - Verbal fluency (phonological and semantic)	fMRI (BOLD contrast)	In SZ, there was a reduction in left hippocampal activity during semantic tasks and a failure to engage the anterior cingulate gyrus during VF tasks.
Adamczyk et. al. 2021 (24)	SZ:30 HC:30	Metaphor comprehension	fMRI-EEG (EC)	Metaphor comprehension in SZ is associated with reduced function and altered effective connectivity in frontotemporoparietal regions.
Sabb et al., 2009	CHR:40 HC:24	Semantic judgments?	fMRI (BOLD contrast)	CHR participants showed increased neural activity in a network of language-associated brain regions, including MPC, left IFG, MTG, and the AC
Dickey el at 2011	SPD:129 HC:138	Speech perception - Prosody Identification	MRI -fMRI	SPD exhibited less efficient STG recruitment during prosody identification and trended towards smaller left STS volumes
Wroblewski et al 2020 (27)	SSD:17 HC:18	Gestures-speech integrations	fMRI (EC)	SSD revealed reduced connectivity in the verbal pathway, specifically from the left MTG to the left STS.
Zhang et al 2016 (30)	SZ:17 HC:21 BD: 23	Semantic judgments	fMRI (FC)	SZ patients exhibited decreased ventral-anterior insula-precuneus/posterior CC functional connectivity.
McIntosh et al., 2008 (45)	SZ: 27 HC: 37 BD:42	Semantic judgments	fMRI (BOLD contrast)	SZ exhibited altered patterns of activity in the dorsal PFC and insula.

**Table 7:** Summary of relevant findings in semantic memory encoding.

Study	Participants	Linguistic measure	Brain Measure	Results
Surbeck et al 2020 (26)	SZ:45 HC: 44	Semantic memory-encoding	DTI	In SSD, semantic processing deficits are linked to compromised integrity in the ventral language stream, specifically the left IFOF.
Rannikko et al 2012 (37)	SZ:57 HC:94	Semantic memory-encoding	MRI (SC)	Lower gray matter volume in the CC, juxtapositional lobule, right STG, and precuneus was found to be associated with semantic memory.
Habets et al 2008 (44)	Psychosis:31 Non-psychotic:32 HC:28	Semantic memory-encoding - Verbal fluency	MRI	VF correlated with grey matter density in striatal nuclei and the thalamus, while concept shifting test scores were associated with cerebellar grey matter density deficits.
Roes et. al. 2021	SZ:40 HC:40	Semantic memory-encoding	fMRI (FC)	SZ exhibited reduced activity in the linguistic processing network, decreased activation in the responding network, and weakened suppression in the DMN.

#### 3.2.1 Semantic

As expected, numerous studies focused on features of meanings. Some specifically delve into the meaning attached to individual words (lexical semantics), while others explore the overarching meaning of discourse within tasks involving language comprehension. In this section, we will review studies focused on the lexicon and the relations among words. In the following section, we will cover those that describe semantics in more general terms, and finally, we will review those that used semantic encoding paradigms.

##### 3.2.1.1 Lexical semantics

We found ten studies addressing comprehensive lexico-semantics paradigms (33, 36, 40, 43, 47, 48, 54, 56, 58, 66). In the following section and in **Table 5**, we review the studies with lateralization analysis, brain activity patterns, and finally, brain connectivity analysis.

Two studies on individuals with first-episode schizophrenia (FES) observed diminished lateralization in brain regions including the IFG and STG. In Bleich-Cohen’s study (43) 12 FES individuals and 17 HC went through a lexical association task to examine the relationship between objects (nouns) and habitual actions (verbs). Participants were instructed to produce a verb that most accurately conveyed what actions they could perform with the specified object they listened. Both groups completed the task without errors. Brain analysis revealed reduced functional lateralization in the IFG and STG. Interestingly, this reduction stemmed from heightened activity in the right hemisphere in FES patients compared to HC, rather than decreased activity in the patients’ left hemispheres (43). In van Veele’s study (40) involving 35 drug-naive FES individuals and 43 HC participants, the Lateralization Index (LI) was examined during three language tasks: paced verb generation, antonym generation, and semantic decision. The results showed a significant reduction in LI among the patient group when compared to the HC group. Notably, this reduction was particularly pronounced in the IFG and STG. The authors did not reported difference in the right left sided activations levels between patients and controls (40). While one study suggests that reduced lateralization is due to increased activation in the right hemisphere, the other only reports differences in the left-right activation ratio.

In five separate studies employing a priming task, distinct patterns of brain activity were observed between individuals with SZ and HC during the presentation of related words, albeit in different brain regions, namely the IFG (BA 44 and BA 45), the fusiform gyrus (BA 37) and the STG (BA 22). In the first study, 12 male participants with SZ and 12 HC participants were exposed to three different conditions: high-connectivity word pairs, low-connectivity word pairs, and unrelated pairs of words. The concept of connectivity assumes that word meanings are organized into semantically associated networks, where, for instance, “food” and “dinner” are highly connected, while “dog” and “house” have low connectivity. In the high connectivity condition, the HC exhibited left hemisphere activity in or near the middle STG and IFG, whereas individuals with SZ showed activity only near the middle STG. In the low connectivity condition, HC demonstrated greater left hemisphere activity, once again in the STG and IFG, whereas individuals with SZ only exhibited activity in the STG. Consequently, the IFG exhibited reduced activity in the patient group while processing related words. Moreover, the SZ group showed less clear within-group activation reductions in response to word pair connectivity in left frontal and temporal regions (48). Employing a similar paradigm, Kuperberg et al. (54) presented three conditions – directly related (e.g., “bell-church”), indirectly related (e.g., “bell-priest”), and unrelated (e.g., “bell-hammer”) to 16 patients with SZ and 20 HC. They found a more pronounced automatic indirect priming effect in the group of patients in the left fusiform gyrus (BA 37). Specifically, neural activity decreased for target words that followed indirectly related (as opposed to unrelated) prime words in the patient group, whereas no such effect was observed with the direct priming effect (54). In another study (SZ:14 and HC:14) a priming paradigm coupled with a visual lexical decision task revealed interaction disparities between priming and modality in the left fronto-temporo-parietal regions and the right AG. They were presented with direct or indirect word pairs, unrelated word pairs, and pseudoword targets presented either visually or auditorily while undergoing fMRI scans. Participants were required to determine whether the target was a real word by pressing one of two buttons. Although behavioral results showed no accuracy differences, word relatedness affected reaction time, with no distinction between the groups. Brain analysis of semantic priming effects unveiled notable distinctions between the two groups. Specifically, patients with SZ exhibited activity enhancement within the right angular gyrus (AG) and precuneus compared to HC. The modality effect displayed variances in activation across several brain regions, including the left MTG, fusiform gyrus, cerebellum, ACC, SFG, and right AG. Generally, control participants demonstrated higher activation levels for crossmodal presentations compared to unimodal ones, and overall, higher activation was observed in patients. Furthermore, significant differences in the interaction between semantic priming and modality were found within the left MTG, SFG, right AG, precuneus, and cerebellum (47). Another study involving lexical decision during scanner (SZ:17, HC:15) used three word-pair conditions: directly and indirectly related, unrelated and non-word. The participants were asked to decide if the words were real words. The behavioral performance of both groups showed no significant differences in priming effects. However, distinct patterns of hemodynamic modulation were observed between the two groups in response to different types of word pairs. Specifically, in relation to directly related word pairs compared to unrelated word pairs, patients with SZ exhibited a pattern of hemodynamic modulation primarily within the left IFG (BA 45 and 47). Similarly, compared to unrelated word pairs, patients with SZ displayed hemodynamic modulation within the left fusiform cortex (BA 37) in response to indirectly related word pairs. Conversely, the HC group exhibited the reverse pattern of hemodynamic modulation within the temporal cortices (56). Similarly, Wilson’s study (66) (SZ:17 and 15 HC) included a lexical decision task during scanner. During the task, the participant had to decide if the target was a real word or not among four conditions categorized as directly related (e.g., salt–pepper), indirectly related (e.g., lion-stripes), unrelated (e.g., star-drill), and word/nonword (e.g., cheek-sporg). The results showed that individuals with SZ exhibited enhanced hemodynamic responses in the left fusiform gyrus (BA 37) and STG (BA 22) for both directly and indirectly related priming. The authors interpreted these results as an increased and longer automatic spreading activation during semantic processing in the group of patients (66).

Using a different paradigm, some authors observed heightened IFG activation and reduced left caudate nucleus activation. The study focused on a semantic association task, where participants (SZ: 20 and HC:20) were required to assess the relationship in meaning between pairs of Chinese characters. Behavioral results indicated no differences in accuracy but slower response times in the patient group. Both groups showed significant activation in the IFG (BA45), caudate nucleus, and fusiform gyrus (BA 37) in the left hemisphere. The patients showed greater activation in the left IFG, reduced activation in the left caudate nucleus and a stronger connection between these areas compared to controls (58).

Finally, using connectivity analysis, Lavigne (36) conducted a study involving 23 patients with SZ (10 experiencing hallucinations, 13 without hallucinations), 22 individuals with BD, and 27 HC and two lexical tasks. The first task involved generating conceptual definitions from an object’s image and corresponding label without verbalizing them. The second task required listening to word definitions. By analyzing functional connectivity, the researchers observed a left-dominant frontal-temporal network encompassing auditory and motor regions associated with the tasks. The network included pars opercularis of the left IFG (BA 44), bilateral STG (BAs 21, 22), FG (BA 37) and supplementary motor area (BA 6). Interestingly, SZ patients with recent hallucinations exhibited heightened connectivity within this network only while listening to the definitions, as compared to patients without hallucinations (36). Woodward studied (33) 30 patients diagnosed with SZ and 30 HC using a semantic integration task with a whole brain connectivity analysis. The task involved selecting the most closely associated word from a set of three options in response to a prompt word (out of the three options, only one was related to the prompt word). Two configurations of multiple demand networks showed similar brain activity in both groups, indicating no significant differences. However, in the semantic integration network, individuals with SZ exhibited reduced functional connectivity compared to those in the HC group. This network involved the left fusiform gyrus (BA 37), left IFG (BA 44 and 47), and left dorsal ACC (BA 32). These findings led the researchers to conclude that disturbances in semantic integration are a key neurocognitive feature of the disorder (33).

##### 3.2.1.2 Semantic meaning

In relation to those studies that addressed semantic meaning, we found three studies (34, 49, 53) that used a semantic task and six studies that utilized more complex paradigms (24, 27, 30, 41, 45, 46) as shown in **Table 6**.

In studies utilizing semantic tasks, the primary brain areas that exhibited differences between patients and controls were the IFG and the STG. In Kubicki’s study (53) 9 patients with SZ underwent a semantic judgment task during scanner. The task consisted of two conditions: a semantic encoding condition, where participants made judgments about whether words were abstract or concrete in meaning, and a shallow, non-semantic encoding condition, where subjects made perceptual judgments about the font size (uppercase or lowercase) of the words. During the semantic encoding condition, individuals with SZ exhibited activation in several brain regions, including the left IFG, left and right middle frontal gyrus (MFG), left posterior STG, left parietal lobe, cingulate gyri, and bilateral occipital lobes. Surprisingly, during the non-semantic encoding condition, patients with SZ activated in the same areas observed during the semantic condition. Furthermore, significant differences emerged when directly comparing the HC group with individuals with SZ under the semantic encoding condition. SZ participants displayed decreased activation in the left IFG and increased activation in the left STG (53). In Royer’s study (34) 31 individuals with SZ and 31 HC participants underwent a functional MRI session while listening to an unknown story as part of a speech-listening paradigm. The findings revealed that individuals with SZ exhibited reduced leftward functional hemispheric lateralization for language compared to the HC group (34). In another study, involving 16 individuals with SZ and 18 HC, participants underwent a passive word reading task while in the MRI scanner. Both groups exhibited activation in the semantic network, which included regions such as the left IFG, left AG, and left MTG. Notably, as the severity of FTD increased, the differences between the semantic networks of patients with SZ and HC became more pronounced. This discrepancy was primarily attributed to a reduced involvement of the left IFG, specifically Brodmann areas 45 and 47 (49). In the same vein, in Kircher´s study (52), during an association task, observed a higher signal change in the left IFG (Brodmann areas 44, 45, 47) compared to reading (52).

In an attempt to understand figurative language, a feature widely altered in patients with SZ, a study involving 30 outpatients diagnosed with SZ and 30 HC, explored effective connectivity during a metaphor comprehension task. The task involved short stories with three possible endings or punchlines: a neutral ending with a literal meaning (NEU), an absurd ending with a meaningless sentence (ABS), and a metaphorical ending with a figurative meaning (MET). During the recognition and elaboration of metaphors (MET vs ABS), individuals with SZ exhibited reduced activation in several brain regions. These regions included the left IFG pars opercularis, left SFG, precuneus (shifted leftward but interhemispheric), as well as the right insula, right frontal-temporal space, right temporal pole (TP) and MTG, and right precentral/postcentral gyri. Furthermore, during the overall processing of metaphors (MET vs NEU), individuals with SZ demonstrated decreased activation in the left caudate, interhemispheric dorsal ACC, left IFG, MFG, SFG, bilateral insula, Heschl gyrus, STG, precuneus, inferior parietal lobule (IPL) including the angular and supramarginal gyri. These findings indicate altered neural activation patterns in individuals with SZ during metaphor comprehension, specifically involving regions associated with language processing and cognitive control (24). In another study, a group of forty individuals at CHR and twenty-four demographically matched HC participated in a fMRI protocol. During the scanner, the participants engaged in a naturalistic discourse processing paradigm. Two conditions were presented to the participants, in which they were asked to judge whether a set of question-answers were congruent or on the same topic (off-topic example: how would you feel in an earthquake? I go to Disneyland in the summer; incongruent example: why are you wearing a raincoat? So I won’t get sad). The study results revealed that CHR participants exhibited increased neural activity in a network of brain regions associated with language processing, including the bilateral medial PFC, the left IFG (BA 44/45 and 47), the left MTG, and the anterior cingulate (BA 24 and 32). Moreover, the individuals who subsequently developed psychosis showed further increased activity in the STG, caudate, and left IFG when compared to the participants who did not develop psychosis (46). In a study conducted by Dickey (41), the focus was on prosody processing in 129 individuals diagnosed with schizotypal personality disorder and 138 HC. Participants performed a prosody identification task, in which they listened to semantically neutral sentences with emotional prosody while undergoing a functional scanning. Contrary to the initial prediction of the authors, the SZ group demonstrated a similar performance and reaction time than the HC group in correctly identifying the conveyed emotion. Both groups exhibited activation in the STG while performing the prosody identification task; however, the HC group showed higher right STG coupling (41). Addressing effective connectivity but in relation to gestures-speech integration, Wroblewski (27) included 17 medicated patients diagnosed with severe and persistent SZ spectrum disorders (SSD) and 18 HC in a study that utilized Dynamic Causal Modelling (DCM) to examine effective connectivity during gestures-speech integrations. During the scanning session, participants were presented with short video sequences featuring an actor performing meaningful gestures while accompanied by spoken sentences in either German or Russian language. Additionally, participants were required to perform a content judgment task, distinguishing between stimuli related to objects or persons. The study’s findings revealed that patients with SSD exhibited significantly weaker coupling from the MTG to the STS. This impairment in connectivity may contribute to the dysfunctional integration of co-verbal, intrinsically meaningful gestures, potentially explaining interpersonal communication difficulties observed in individuals with SSD (27). In Zhang´s study (30) involving 23 patients with BD, 17 patients with SZ, and 21 HC, participants engaged in a self-reflection task that included three conditions: self-reflection, close other-reflection, and semantic control. During the task, participants had to decide whether a sentence containing a trait word applied to themselves (self-reflection) or to a close individual (close other-reflection). When comparing the close other-reflection condition to the semantic control condition, individuals with SZ exhibited reduced functional connectivity between the ventral-anterior insula and the precuneus/posterior cingulate cortex (CC). In contrast, HC showed increased functional connectivity in the same brain regions during the close other-reflection condition (30). Finally, McIntosh (45) conducted a study involving 27 patients diagnosed with SZ and 37 HC. The participants were asked to complete the verbal initiation section of the Hayling Sentence Completion Test while inside the scanner. They presented sentences in which the final word was missing, and they were required to generate an appropriate word to complete the sentence mentally. Once they had formulated their response, they pressed a button. The study’s findings indicated differences between patients and controls in the dorsal PFC and insula (45).

It’s noteworthy that, up to this point, the most significant differences between patients and controls were found in the IFG and STG and occasionally in the vATL. However, with the integration of information from various elements and increased semantic complexity, it is observed that while some areas, like the IFG, continue to show differences, additional significant differences also appear in the angular gyrus, supramarginal gyrus, precuneus, and MTG.

##### 3.2.1.3 Semantic memory encoding

Regarding the studies of semantic encoding, we found six studies all in chronic patients as shown in **Table 7**. A study with SZ (N= 45) patients compared with HC (N=44) found a decrease in the integrity of the IFOF and that the uncinate fasciculus was inversely correlated with semantic impairments (26). Another investigation using the California verbal learning test in 57 SZ patients compared with 94 HC, revealed that even after accounting for illness duration, sex, and total gray matter, lower gray matter volume in the CC, juxtapositional lobule, right STG, and precuneus was linked to poorer verbal memory (37). Habets’ (39) study demonstrated a negative association between Concept Shifting Test scores and gray matter density in the left cerebellar hemisphere, observed in patients and relatives. Lastly, with a functional approach, a study involving 40 individuals diagnosed with SZ and 40 HC using a Paired Associates Encoding Task during the scanner revealed the presence of three distinct functional networks throughout the entire brain that were actively engaged during the process of paired associates learning. These networks include a responding network, a language/attention network (left MTG, left FG, left MFG, IFC, and dorsal ACC), and the default mode network (DMN) encompassing the medial cingulate and precuneus cortex, as well as bilateral posterior temporal regions. Notably, individuals with SZ exhibited hypoactivity within the semantic network and inefficiency in suppressing the DMN. The author suggests that the patient’s capacity to employ semantic strategies during episodic memory encoding may be limited (23).

## 4 Discussion

In this review, we have compiled studies focusing on examining language networks in individuals with SZ using a sign-based approach rather than focusing on symptoms of thought disorders. We observed recurring and consistent patterns in some studies, while differences emerged in others. Overall, the findings related to the production of language suggested different brain hemodynamic responses in the prefrontal and temporal cortex and reduced functional connectivity between the medial prefrontal cortex and subcortical regions during verbal fluency tasks; different patterns of activation about the amount of speech and syntactic complexity and; related to semantic tasks there was a covariation between linguistic features and the brain activity/connectivity of the temporal and frontal cortex. Regarding comprehension of language, the findings showed less left lateralization in the group of patients, different activation in the IFG, AG, FG, precuneus and STG (some studies observed reduction while others enhanced) in lexical tasks; in the semantic tasks, the studies observed different patterns of activation in IFG, medial PFC, ACC, precuneus, STG and MTG; and in the memory encoding tasks the studies showed reduced gray matter in the group of patients and decrease activity in the DMN. We will now delve into the methodological distinctions that may contribute to these studies’ discrepancies.

Concerning Verbal fluency, several studies have examined the relationship between performance and brain measures. Regarding structural brain measures, in UHR individuals, two studies (39, 42) observed that the verbal fluency deficit was related to reduced grey matter density and lower left lateralization of pars triangularis. The reduced grey matter density was also observed in FES patients but in basal ganglia and thalamus regions (44). Notably, Habets (44) also found reduced grey matter in frontal, temporal and insular areas, but they were not related to the verbal fluency task. In Psychosis patients, two studies found tract differences in the left superior longitudinal fasciculus, inferior longitudinal fasciculus and inferior frontal-occipital fasciculus (28, 35). These results suggest that the grey-and-white matter of the language network predicts, to some extent, better verbal fluency performance. Concerning the functional measures, all the studies (29, 31, 32, 38, 52) except for one (55) observed reduced brain activity in relation to verbal fluency tasks. This difference in results is attributable to the fact that Ragland (55) included switching conditions to increase the cognitive demand of the task. The findings indicate that in over-learned conditions, the neural processes involved in lexical retrieval and word generation remain relatively unaffected in individuals with schizophrenia and that poorly modulated neural response during standard fluency tasks is secondary to well-documented deficits in cognitive control. The areas involved were IFG, STG, bilateral PFC, brainstem, insula, and the left hippocampus. This is intuitively plausible, considering that deficiencies in strategic memory typically correlate with frontal lobe dysfunction or subcortical structures associated with circuits involving the frontal lobe. Furthermore, even though the verbal fluency task is straightforward to apply, the analysis of the studies we reviewed lacks adequacy in explaining the intricate retrieval system. Indeed, incorporating new approaches, such as computational linguistics, is essential to gain a detailed understanding of this type of data (67).

In the domain of semantics, specifically concerning the productive dimension, three articles employed NLP techniques to analyze language samples. Two articles correlated these linguistic metrics with resting-state brain activity, while the third article integrated an additional task during the scanning process. It is not surprising that the results among these studies were inconsistent, given the diverse nature of the experiments. Tagamets (57) utilized NLP metrics to establish correlations with brain activity; however, participants were engaged in a task during the scanning process where they had to monitor whether the stimulus matched the previous one in terms of spelling and sound. Their findings revealed a cluster of activity in the bilateral SFG, right supramarginal gyrus, right FG, right STP, and right culmen/vermis. In the other two studies, participants were in a resting state during the scanning process. They used different types of speech samples (autobiographic life changes versus picture descriptions), yet the results were consistent. Alonso’s study (22) suggests that lexical impoverishment observed in individuals with SZ may be attributed to increased self-inhibition in the IFG and vATL, possibly stemming from reduced synaptic gain and diminished precision of locally generated neural activity (22). Similarly, In Haas’s (25) study, semantic coherence measured through LSA was related to functional connectivity between the temporal and prefrontal semantic hubs. We hypothesize that the differences in the cognitive demand during scanner, such as homophones and homographs matching (25) versus resting state (22), may play a crucial role in accounting for these disparities. While none of the tasks is ideally suited for addressing the relation between lexical selection and brain connectivity, the evidence indicates a striking similarity in the connectivity of the semantic network between the active semantic task and the resting-state data (68). In other words, although it may be less goal-directed and stimulus-driven, the process of conceptualization is almost unavoidable during the resting state.

Regarding syntax, we only found three studies, and two were from the same author. Kircher’s (50, 51) studies showed that the HC group showed correlations between the number of complex sentences produced and activation in the posterior portion of the right MTG and the left SFG. In contrast, these correlations were absent in the patient group. Liang observed that the mean length of T-units (complexity) in the speech and the lexical cohesion were related to the cortical thickness (59). Since the nature of the brain measure is quite different, is not possible to compare the results. Additionally, there are variations in the method of measuring syntactic complexity. Kirchner differentiated between simple and complex sentences based on clauses, while Liang utilized T units of the sentences.

Regarding comprehensive lexical tasks, most studies focused on conventional language regions, notably the IFG and the STG. The only two studies addressed lateralization of language-related brain activation and observed consistently less lateralization in the group of FES (40, 43). Regarding the comparison of brain activity, Han (48) observed enhanced IFG activity with a lexical decision task. At the same time, Chen (47) found less activity in the same brain region in a lexical association with Chinese characters task. In this same line, Kuperberg’s (54) study localized the N400 priming effect spatially in the left temporal fusiform cortex. These results may suggest an elevated and prolonged automatic spreading activation during the semantic processing in the patient group. Some studies found increased connectivity or activity, while others found the opposite. The variances observed could potentially originate from the specific task employed in each study or the clinical characteristics of the sample. For example, Woodward (33) found decreased connectivity, while Lavigne (36) found higher connectivity. However, the first compared SZ with HC, whereas the second compared SZ patients with and without hallucinations. Overall, using a priming task, several studies observed heightened hemodynamic responses among the IFG, FG and STG.

In the semantic meaning paradigms, the results were not consistent in the studies. Using passive listening, Royer’s study (34) identified differences in the leftward functional laterality indices between patients and controls. Kubicki’s (53) study revealed decreased activation in the left IFG and increased activation in the left STG during a semantic judgment between abstract and concrete words. While Horn’s (49) study observed reduced left IFG activation in the patient’s group during passive word reading. The difference in the STG activation between these results could be explained by the nature of the task and the brain recruitment. Indeed, the STG is one of the key candidates for abstract concept processing (69), while semantic processing during passive word reading is more related to MTG (70). The consistent pattern of reduced activity in the IFG is worth noting, with evidence pointing towards the IFG’s role in controlled semantic tasks.

The remaining studies categorized as semantic comprehension paradigms involved higher cognitive demands, resulting in more extensive brain engagement. In Sabb’s (46) experiment, participants were tasked with monitoring whether the auditory stimuli belonged to the same topic and whether they were congruent, thus encompassing a broad range of semantic processing. They observed an increased activation in bilateral medial PFC, left IFG, left MTG, and ACC in the CHR group. Moreover, the CHR psychosis converters exhibited heightened neural activity in left IFG, caudate and STG regions that may serve as a potential marker for identifying those who are at a higher risk of developing psychosis.

Adamczyk (24) utilized a cognitively demanding task focused on metaphor comprehension and found decreased activation in the left caudate, interhemispheric dorsal ACC, left IFG, mFG, and SFG, left insula, Heschl gyrus, STG, precuneus, and inferior parietal lobule including the angular and supramarginal gyri. Zhang (30) used a self-reflection task and observed reduced functional connectivity between the ventral-anterior insula and the precuneus/posterior CC when engaging in close other-reflection tasks. Here emerges a pattern of heightened engagement of cognitive domains associated with complex tasks involving the brain’s activation of the precuneus and insula.

Dickey (41) explored prosodic integration in schizotypal personality disorder individuals and observed differences in the right/left STG coupling compared to HC. Wroblewski (27) studied gestural processing and observed significantly weaker coupling from the MTG to the STS in SSD individuals. Finally, McIntosh (45) employed a task with a more executive profile, specifically the Hayling task, in which they observed a different activity pattern in the dorsal PFC and insula (45).

Some differences in the results may be due to the broad sense in which the concept of “semantics” is used to describe properties of the discourse of SZ patients. In fact, some studies focus on the overall meaning of discourse (22, 24, 25, 27, 30, 34, 45, 46, 49, 57, 66), while others focus on lexical meanings (33, 36, 40, 43, 47, 48, 53, 56, 58). This difference is relevant because language phenomena at both levels of analysis are different. In Linguistics terms, discourse’s overall meaning, or semantics, emerges from the interplay among various language levels, including phonological, morphological, lexical, syntactic, and interclausal components. These levels interact with each other and elements specific to the cultural and situational context in which the discourse takes place (71). The text, as a unit of meaning, conveys semantically coherent and lexically and syntactically cohesive ideas aimed towards a specific goal, influenced by contextual factors such as the speakers’ task (conversation, discussion, presentation) and the discourse modality [narration, explanation, argumentation, instruction (72)]. Within this framework, studies explore preservation, semantic distance between text elements (22, 25), discourse generation based on text comprehension (24, 46), semantic judgment (30), and semantic coherence (57). On the other hand, lexical meanings, or lexical semantics, pertain to the meanings of individual lexical units within a language and the relationships between them within specific lexical-semantic fields. Psycholinguistic terms also involves speakers’ ability to construct and retrieve lexical units from their mental lexicon. Within this context, studies investigate lexical decision tasks, associations between lexical units (33), meaning generation (36), judgment of lexical units (40, 47, 53, 56, 58), and generation of new lexical units (43).

Similarly, in neurolinguistic terms, activation patterns vary based on cognitive demands. For instance, tasks associated with lexical selection heavily engage the inferior frontal gyrus (IFG), while those requiring greater executive functions recruit more prefrontal areas. Similarly, more intricate processes like metaphor processing involve multiple brain regions. Notably, prosodic processing exhibits a more substantial reliance on the right hemisphere. The significance of the superior temporal gyrus (STG) in semantic processing is also prominently observed. In summary, the results indicate that the language network, particularly the semantic network (sometimes more narrow and at others more broad), manifests distinct activation patterns in patients.

Finally, when examining memory encoding paradigms, it was found that individuals with SSD had lower gray matter volume in the CC, juxtapositional lobule, right STG, and precuneus in one study (46) and lower cerebellar gray matter density in another (44) in relation to a verbal learning test.

The variations in the results can be attributed to the group comparisons. In the first study, SZ individuals were compared with a control group, whereas in the second study, SZ individuals were compared with non-psychotic first-degree relatives. Moreover, this semantic processing was functionally related to decreased linguistic processing network activity and reduced DMN suppression (23). This is a plausible assertion, given that both the DMN and the language network are key candidates for neural markers of schizophrenia.

There are some limitations in the results of this work. In our review of the language network, we made an effort to explore its widely defined components based on existing literature. Several studies pre-defined specific brain areas, resulting in variations in the comparison of brain regions across patients and HC. For instance, certain areas of the language network, such as the IFG, consistently exhibit differences, however, it is one of the few areas that is considered in all analyses. Therefore, not all brain areas were consistently examined in all studies included in our analysis.

Furthermore, another constraint lies in the fact that only a limited number of studies have employed machine learning models for analyzing language features. Computational language models have comparative advantages over alternative other approaches to describe linguistic data. A specific illustration of their utility can be observed in the language model employed to extract all the metadata from this very review. Nevertheless, the studies of this review predominantly explore language variables using experimental paradigms designed to isolate one of the variables by controlling the others. However, extracting variables from language phenomena in this manner is highly intricate and results in a significant loss of information. Language is inherently contextual, and a substantial portion of the meaning is intricated in the different layers, levels of language with multiple micro processes in this case.

In that scenario, a large and structured set of texts -oral or written corpus- produced in ecological conditions allows researchers to test existing neuro and psycholinguistics theories and models, assessing their validity with data based on contextual language, and eventually, develop new theoretical frameworks to explain them. More specifically, the metrics of the language structure not only provide information about its use, also allow relating these occurrences with neurobiological evidence. Based on the limitations identified in this literature review, we propose certain guidelines for future research in this field. Examining larger networks involved in language processing -or networks that work together (eg. DMN and Salience Network)- can provide a more comprehensive understanding of the neural mechanisms underlying linguistic/semantic processing in individuals with SSD.

Moreover, extensive evidence exists pointing towards discernible patterns in the speech and communication of individuals with schizophrenia (SZ). Similarly, brain patterns have been identified in association with SZ. However, to date, no specific biomarkers have been identified for SZ, either in linguistics or neuroimaging. It is worth noting that language models have demonstrated the capability to predict brain measurements (73). Thus, there is a pressing need to integrate and combine these models with brain metrics, not solely for classification purposes, but also to gain a qualitative understanding of the language processes involved in SZ. This understanding can then serve as a foundation for identifying potential targets for neuromodulation interventions. In summary, future research should integrate language models and neuroimaging while considering broader brain networks in analyses. By following these guidelines, we can advance our knowledge of language processing in individuals with severe mental illness and enhance the validity and generalizability of findings in this field.

Beyond the limitations of the review’s results, there are also inherent limitations within the review itself. Firstly, its generalizability is constrained as the findings primarily pertain to SZ, potentially limiting applicability to other psychiatric or neurological conditions. We acknowledge the importance of transdiagnosis research, however most of the results were comparing SZ patients with controls. Additionally, an over-reliance on neuroimaging might overshadow other crucial aspects of language processing in SZ. The inclusion of additional techniques, such as EEG, was impractical due to scope limitations and this would suffice to constitute a new comprehensive review. Furthermore, there is a possibility of bias in interpretation stemming from the backgrounds and prior studies of the review’s authors, potentially influencing study selection and interpretation. Lastly, given the rapidly evolving nature of the fields of neuroimaging and computational linguistics, certain sections of the review may require updating in the coming years.

## Data availability statement

The original contributions presented in the study are included in the article/**Supplementary Material**. Further inquiries can be directed to the corresponding author.

## Author contributions

MA-S, PA-F, and MO contributed to conception and design of the study. JP performed the search. MA-S, LZ-R, and PA-F analyzed the results. MA-S, PA-F, MO, LZ-R, and AC wrote the manuscript. All authors contributed to manuscript revision, read, and approved the submitted version.

## Glossary

**Table:**

AG	Angular Gyrus
BD	Bipolar Disorder
DMN	Default Mode Network
HC	Healthy Controls
IPL	Inferior Parietal Lobule
SVF	Semantic Verbal Fluency
ACC	Anterior Cingulate Cortex
AD	Axial Diffusivity
BERT	Bidirectional Encoder Representations from Transformers
CC	Cingulate Cortex
CEN	Central Executive Network
CHR	Clinical High-Risk
SZ	Schizophrenia
DCM	Dynamic Causal Modelling
FA	Fractional Anisotropy
FES	First Episode Schizophrenia
FG	Fusiform Gyrus
fMRI	Functional Magnetic Resonance Imaging
fNIRS	Functional Near-Infrared Spectroscopy
FTD	Formal Thought Disorder
gPPI	Generalized Psychophysiological Interaction
IFG	Inferior Frontal Gyrus
IFOF	Inferior Fronto-Occipital Fasciculus
ILF	Inferior Longitudinal Fasciculus
IOG	Inferior Occipital Gyrus
LI	Lateralization Index
LSA	Latent Semantic Analysis
MFG	Middle Frontal Gyrus
mFG	Medial Frontal Gyrus
MTG	Middle Temporal Gyrus
MWF	Myelin Water Fraction
PA	Paranoia
PFC	Prefrontal Cortex
PRISMA	Preferred Reporting Items for Systematic Reviews and Meta-Analyses
PS	Psychoticism
PVF	Phonological Verbal Fluency
RSN	Resting State Networks
SFG	Superior Frontal Gyrus
SMA	Supplementary Motor Area
SN	Salience Network
SQuAD	Stanford Question Answering Dataset
SSD	Schizophrenia Spectrum Disorders
STG	Superior Temporal Gyrus
STROBE	Strengthening the Reporting of Observational Studies in Epidemiology
STS	Superior Temporal Sulcus
SZ	Schizophrenia
TLC	Thought, Language and Communication
TLI	Thought and Language Index
UHR	Ultra-High Risk
vATL	Ventral Anterior Temporal Lobe.

## References

[1] Kraepelin E . Dementia praecox and paraphrenia. Livingstone. (1919).

[2] Covington MA He C Brown C Naçi L McClain JT Fjordbak BS . Schizophrenia and the structure of language: The linguist’s view. Schizophr Res. (2005) 77:85–98. doi: 10.1016/j.schres.2005.01.016 16005388

[3] Kuperberg GR Kreher DA Ditman T . What can Event-related Potentials tell us about language, and perhaps even thought, in schizophrenia? Int J Psychophysiol. (2010) 75:66–76. doi: 10.1016/j.ijpsycho.2009.09.005 19765622 PMC3136365

[4] Moskowitz A Heim G . Eugen bleuler’s dementia praecox or the group of schizophrenias (1911): A centenary appreciation and reconsideration. Schizophr Bull. (2011) 37(3):471–9. doi: 10.1093/schbul/sbr016 PMC3080676 21505113

[5] Andreasen NC . Thought, language, and communication disorders: I. Clinical assessment, definition of terms, and evaluation of their reliability. Arch Gen Psychiatry. (1979) 36:1315. doi: 10.1001/archpsyc.1979.01780120045006 496551

[6] Liddle PF Ngan ETC Caissie SL Anderson CM Bates AT Quested DJ . Thought and Language Index: an instrument for assessing thought and language in schizophrenia. Br J Psychiatry. (2002) 181:326–30. doi: 10.1192/bjp.181.4.326 12356660

[7] Wensing T Cieslik EC Müller VI Hoffstaedter F Eickhoff SB Nickl-Jockschat T . Neural correlates of formal thought disorder: An activation likelihood estimation meta-analysis. Hum Brain Mapp. (2017) 38:4946–65. doi: 10.1002/hbm.23706 PMC5685170 28653797

[8] Cavelti M Kircher T Nagels A Strik W Homan P . Is formal thought disorder in schizophrenia related to structural and functional aberrations in the language network? A systematic review of neuroimaging findings. Schizophr Res. (2018) 199:2–16. doi: 10.1016/j.schres.2018.02.051 29510928

[9] Sumner PJ Bell IH Rossell SL . A systematic review of task-based functional neuroimaging studies investigating language, semantic and executive processes in thought disorder. Neurosci Biobehav Rev. (2018) 94:59–75. doi: 10.1016/j.neubiorev.2018.08.005 30142368

[10] Chen J Wensing T Hoffstaedter F Cieslik EC Müller VI Patil KR . Neurobiological substrates of the positive formal thought disorder in schizophrenia revealed by seed connectome-based predictive modeling. NeuroImage Clin. (2021) 30:102666. doi: 10.1016/j.nicl.2021.102666 34215141 PMC8105296

[11] Murphy J Baxevanis AD . Searching the NCBI databases using entrez. Curr Protoc Hum Genet. (2004) 40::6.10.1–21. doi: 10.1002/0471142905.hg0610s40 18428394

[12] Canese K Jentsch J Myers C . PubMed: the bibliographic database. In: The NCBI Handbook. Bethesda, MD: National Center for Biotechnology Information; 2002. Available at: http://www.ncbi.nlm.nih.gov/books/NBK21101/. Updated August 13, 2003.

[13] Fontelo P Liu F Ackerman M . ask MEDLINE: a free-text, natural language query tool for MEDLINE/PubMed. BMC Med Inform Decis Mak. (2005) 5:5. doi: 10.1186/1472-6947-5-5 15760470 PMC1079856

[14] Cock PJA Antao T Chang JT Chapman BA Cox CJ Dalke A . Biopython: freely available Python tools for computational molecular biology and bioinformatics. Bioinformatics. (2009) 25:1422–3. doi: 10.1093/bioinformatics/btp163 PMC2682512 19304878

[15] Bordeson A Nylander I . A case of severe developmental motor aphasia with psychotic symptoms successfully treated by speech therapy. Acta Paedopsychiatr. (1963) 30:59–70. 14013990

[16] Devlin J Chang MW Lee K Toutanova K . BERT: Pre-training of Deep Bidirectional Transformers for Language Understanding (2018). Available online at: https://arxiv.org/abs/1810.04805.

[17] Guven ZA Unalir MO . Natural language based analysis of SQuAD: An analytical approach for BERT. Expert Syst Appl. (2022) 195:116592. doi: 10.1016/j.eswa.2022.116592

[18] Jin Q Dhingra B Liu Z Cohen W Lu X . (2019). PubMedQA: A dataset for biomedical research question answering. Proceedings of the 2019 Conference on Empirical Methods in Natural Language Processing and the 9th International Joint Conference on Natural Language Processing (EMNLP-IJCNLP). pp. 2567–77. Hong Kong, China: Association for Computational Linguistics. Available at: https://www.aclweb.org/anthology/D19-1259.

[19] Mohamed Hassan HA Marengo E Nutt W . A BERT-Based Model for Question Answering on Construction Incident Reports. . In: Rosso P Basile V Martínez R Métais E Meziane F , editors. Natural language processing and information systems. NLDB 2022. Lecture notes in Computer Science. Vol. 13286. Cham: Springer. (2022). Available at: 10.1007/978-3-031-08473-7_20.

[20] Moher D . Preferred reporting items for systematic reviews and meta-analyses: the PRISMA statement. Ann Intern Med. (2009) 151:264. doi: 10.7326/0003-4819-151-4-200908180-00135 19622511

[21] Vandenbroucke JP Von Elm E Altman DG Gøtzsche PC Mulrow CD Pocock SJ . Strengthening the reporting of observational studies in epidemiology (STROBE): explanation and elaboration. Int J Surg. (2014) 12:1500–24. doi: 10.1016/j.ijsu.2014.07.014 25046751

[22] Alonso-Sánchez MF Limongi R Gati J Palaniyappan L . Language network self-inhibition and semantic similarity in first-episode schizophrenia: A computational-linguistic and effective connectivity approach. Schizophr Res. (2023) 259:97–103. doi: 10.1016/j.schres.2022.04.007 35568676

[23] Roes MM Chinchani AM Woodward TS . Reduced functional connectivity in brain networks underlying paired associates memory encoding in schizophrenia. Biol Psychiatry Cognit Neurosci Neuroimaging. (2023) 8:61–70. doi: 10.1016/j.bpsc.2021.07.003 34303847

[24] Adamczyk P Jáni M Ligeza TS Płonka O Błądziński P Wyczesany M . On the role of bilateral brain hypofunction and abnormal lateralization of cortical information flow as neural underpinnings of conventional metaphor processing impairment in schizophrenia: an fMRI and EEG study. Brain Topogr. (2021) 34:537–54. doi: 10.1007/s10548-021-00849-x PMC8195899 33973137

[25] Haas SS Doucet GE Garg S Herrera SN Sarac C Bilgrami ZR . Linking language features to clinical symptoms and multimodal imaging in individuals at clinical high risk for psychosis. Eur Psychiatry. (2020) 63:e72. doi: 10.1192/j.eurpsy.2020.73 32778184 PMC7443790

[26] Surbeck W Hänggi J Scholtes F Viher PV Schmidt A Stegmayer K . Anatomical integrity within the inferior fronto-occipital fasciculus and semantic processing deficits in schizophrenia spectrum disorders. Schizophr Res. (2020) 218:267–75. doi: 10.1016/j.schres.2019.12.025 31948896

[27] Wroblewski A He Y Straube B . Dynamic Causal Modelling suggests impaired effective connectivity in patients with schizophrenia spectrum disorders during gesture-speech integration. Schizophr Res. (2020) 216:175–83. doi: 10.1016/j.schres.2019.12.005 31882274

[28] Vanes L Mouchlianitis E Barry E Patel K Wong K Shergill S . Cognitive Correlates of abnormal myelination in psychosis. Schizophr Bull. (2019) 45:S325–5. doi: 10.1093/schbul/sbz020.594 PMC6435797 30914748

[29] Vandevelde A Leroux E Delcroix N Dollfus S . Fronto-subcortical functional connectivity in patients with schizophrenia and bipolar disorder during a verbal fluency task. World J Biol Psychiatry. (2018) 19:S124–32. doi: 10.1080/15622975.2017.1349339 28669318

[30] Zhang L Vander Meer L Opmeer EM Marsman JBC Ruhé HG Aleman A . Altered functional connectivity during self- and close other-reflection in patients with bipolar disorder with past psychosis and patients with schizophrenia. Neuropsychologia. (2016) 93:97–105. doi: 10.1016/j.neuropsychologia.2016.09.020 27693668

[31] Iwashiro N Koike S Satomura Y Suga M Nagai T Natsubori T . Association between impaired brain activity and volume at the sub-region of Broca’s area in ultra-high risk and first-episode schizophrenia: A multi-modal neuroimaging study. Schizophr Res. (2016) 172:9–15. doi: 10.1016/j.schres.2016.02.005 26873807

[32] Holper L Aleksandrowicz A Müller M Ajdacic-Gross V Haker H Fallgatter AJ . Brain correlates of verbal fluency in subthreshold psychosis assessed by functional near-infrared spectroscopy. Schizophr Res. (2015) 168:23–9. doi: 10.1016/j.schres.2015.07.043 26277535

[33] Woodward TS Tipper CM Leung AL Lavigne KM Sanford N Metzak PD . Reduced functional connectivity during controlled semantic integration in schizophrenia: A multivariate approach: Reduced Functional Connectivity. Hum Brain Mapp. (2015) 36:2948–64. doi: 10.1002/hbm.v36.8 PMC6869836 26014890

[34] Royer C Delcroix N Leroux E Alary M Razafimandimby A Brazo P . Functional and structural brain asymmetries in patients with schizophrenia and bipolar disorders. Schizophr Res. (2015) 161:210–4. doi: 10.1016/j.schres.2014.11.014 25476118

[35] Hatton SN Lagopoulos J Hermens DF Hickie IB Scott E Bennett MR . White matter tractography in early psychosis: clinical and neurocognitive associations. J Psychiatry Neurosci. (2014) 39:417–27. doi: 10.1503/jpn.130280 PMC4214876 25111788

[36] Lavigne KM Rapin LA Metzak PD Whitman JC Jung K Dohen M . Left-dominant temporal-frontal hypercoupling in schizophrenia patients with hallucinations during speech perception. Schizophr Bull. (2015) 41:259–67. doi: 10.1093/schbul/sbu004 PMC4266284 24553150

[37] Rannikko I Paavola L Haapea M Huhtaniska S Miettunen J Veijola J . Verbal learning and memory and their associations with brain morphology and illness course in schizophrenia spectrum psychoses. J Clin Exp Neuropsychol. (2012) 34:698–713. doi: 10.1080/13803395.2012.668875 22512417

[38] Allen P Luigjes J Howes OD Egerton A Hirao K Valli I . Transition to psychosis associated with prefrontal and subcortical dysfunction in ultra high-risk individuals. Schizophr Bull. (2012) 38:1268–76. doi: 10.1093/schbul/sbr194 PMC3494046 22290265

[39] Meijer JH Schmitz N Nieman DH Becker HE Van Amelsvoort TAMJ Dingemans PM . Semantic fluency deficits and reduced grey matter before transition to psychosis: A voxelwise correlational analysis. Psychiatry Res Neuroimaging. (2011) 194:1–6. doi: 10.1016/j.pscychresns.2011.01.004 21831606

[40] Van Veelen NMJ Vink M Ramsey NF Sommer IEC Van Buuren M Hoogendam JM . Reduced language lateralization in first-episode medication-naive schizophrenia. Schizophr Res. (2011) 127:195–201. doi: 10.1016/j.schres.2010.12.013 21237617

[41] Dickey CC Morocz IA Minney D Niznikiewicz MA Voglmaier MM Panych LP . Factors in sensory processing of prosody in schizotypal personality disorder: An fMRI experiment. Schizophr Res. (2010) 121:75–89. doi: 10.1016/j.schres.2010.03.008 20362418 PMC2905482

[42] Bhojraj TS Francis AN Rajarethinam R Eack S Kulkarni S Prasad KM . Verbal fluency deficits and altered lateralization of language brain areas in individuals genetically predisposed to schizophrenia. Schizophr Res. (2009) 115:202–8. doi: 10.1016/j.schres.2009.09.033 PMC4841274 19840895

[43] Bleich-Cohen M Hendler T Kotler M Strous RD . Reduced language lateralization in first-episode schizophrenia: An fMRI index of functional asymmetry. Psychiatry Res Neuroimaging. (2009) 171:82–93. doi: 10.1016/j.pscychresns.2008.03.002 19185468

[44] Habets P Krabbendam L Hofman P Suckling J Oderwald F Bullmore E . Cognitive performance and grey matter density in psychosis: functional relevance of a structural endophenotype. Neuropsychobiology. (2008) 58:128–37. doi: 10.1159/000182889 19088490

[45] McIntosh AM Whalley HC McKirdy J Hall J Sussmann JED Shankar P . Prefrontal function and activation in bipolar disorder and schizophrenia. Am J Psychiatry. (2008) 165:378–84. doi: 10.1176/appi.ajp.2007.07020365 18198268

[46] Sabb FW Van Erp TGM Hardt ME Dapretto M Caplan R Cannon TD . Language network dysfunction as a predictor of outcome in youth at clinical high risk for psychosis. Schizophr Res. (2010) 116:173–83. doi: 10.1016/j.schres.2009.09.042 PMC2818263 19861234

[47] Sass K Heim S Sachs O Straube B Schneider F Habel U . Neural correlates of semantic associations in patients with schizophrenia. Eur Arch Psychiatry Clin Neurosci. (2014) 264:143–54. doi: 10.1007/s00406-013-0425-0 23880958

[48] Han SD Nestor PG Hale-Spencer M Cohen A Niznikiewicz M McCarley RW . Functional neuroimaging of word priming in males with chronic schizophrenia. NeuroImage. (2007) 35:273–82. doi: 10.1016/j.neuroimage.2006.11.029 PMC1852450 17215145

[49] Horn H Jann K Federspiel A Walther S Wiest R Müller T . Semantic network disconnection in formal thought disorder. Neuropsychobiology. (2012) 66:14–23. doi: 10.1159/000337133 22797273

[50] Kircher TTJ Oh TM Brammer MJ McGuire PK . Neural correlates of syntax production in schizophrenia. Br J Psychiatry. (2005) 186:209–14. doi: 10.1192/bjp.186.3.209 15738501

[51] Kircher TTJ Liddle PF Brammer MJ Williams SCR Murray RM McGUIRE PK . Reversed lateralization of temporal activation during speech production in thought disordered patients with schizophrenia. Psychol Med. (2002) 32:439–49. doi: 10.1017/S0033291702005287 11989989

[52] Kircher T Whitney C Krings T Huber W Weis S . Hippocampal dysfunction during free word association in male patients with schizophrenia. Schizophr Res. (2008) 101:242–55. doi: 10.1016/j.schres.2008.02.003 18356025

[53] Kubicki M . An fMRI study of semantic processing in men with schizophrenia. NeuroImage. (2003) 20:1923–33. doi: 10.1016/S1053-8119(03)00383-5 PMC2806220 14683698

[54] Kuperberg GR Weber K Delaney-Busch N Ustine C Stillerman B Hämäläinen M . Multimodal neuroimaging evidence for looser lexico-semantic networks in schizophrenia:Evidence from masked indirect semantic priming. Neuropsychologia. (2019) 124:337–49. doi: 10.1016/j.neuropsychologia.2018.10.024 PMC6453534 30391565

[55] Ragland JD Moelter ST Bhati MT Valdez JN Kohler CG Siegel SJ . Effect of retrieval effort and switching demand on fMRI activation during semantic word generation in schizophrenia. Schizophr Res. (2008) 99:312–23. doi: 10.1016/j.schres.2007.11.017 PMC2383319 18155880

[56] Kuperberg GR Deckersbach T Holt DJ Goff D West WC . Increased temporal and prefrontal activity in response to semantic associations in schizophrenia. Arch Gen Psychiatry. (2007) 64:138. doi: 10.1001/archpsyc.64.2.138 17283282

[57] Tagamets MA Cortes CR Griego JA Elvevåg B . Neural correlates of the relationship between discourse coherence and sensory monitoring in schizophrenia. Cortex. (2014) 55:77–87. doi: 10.1016/j.cortex.2013.06.011 23969195 PMC3899100

[58] Chen PJ Fan LY Hwang TJ Hwu HG Liu CM Chou TL . The deficits on a cortical–subcortical loop of meaning processing in schizophrenia. NeuroReport. (2013) 24:147–51. doi: 10.1097/WNR.0b013e32835df562 23324649

[59] Liang L Silva AM Jeon P Ford SD MacKinley M Théberge J . Widespread cortical thinning, excessive glutamate and impaired linguistic functioning in schizophrenia: A cluster analytic approach. Front Hum Neurosci. (2022) 16:954898. doi: 10.3389/fnhum.2022.954898 35992940 PMC9390601

[60] Van Essen DC Smith SM Barch DM Behrens TEJ Yacoub E Ugurbil K . The WU-minn human connectome project: an overview. NeuroImage. (2013) 80:62–79. doi: 10.1016/j.neuroimage.2013.05.041 23684880 PMC3724347

[61] Tournier JD Smith R Raffelt D Tabbara R Dhollander T Pietsch M . MRtrix3: A fast, flexible and open software framework for medical image processing and visualisation. NeuroImage. (2019) 202:116137. doi: 10.1016/j.neuroimage.2019.116137 31473352

[62] Wasserthal J Neher P Maier-Hein KH . TractSeg - Fast and accurate white matter tract segmentation. NeuroImage. (2018) 183:239–53. doi: 10.1016/j.neuroimage.2018.07.070 30086412

[63] Desikan RS Ségonne F Fischl B Quinn BT Dickerson BC Blacker D . An automated labeling system for subdividing the human cerebral cortex on MRI scans into gyral based regions of interest. NeuroImage. (2006) 31:968–80. doi: 10.1016/j.neuroimage.2006.01.021 16530430

[64] Destrieux C Fischl B Dale A Halgren E . Automatic parcellation of human cortical gyri and sulci using standard anatomical nomenclature. NeuroImage. (2010) 53:1–15. doi: 10.1016/j.neuroimage.2010.06.010 20547229 PMC2937159

[65] Glasser M Coalson TS Robinson EC Hacker CD Harwell J Yacoub E . A multi-modal parcellation of human cerebral cortex. Nature. (2016) 536:171–8. doi: 10.1038/nature18933 PMC4990127 27437579

[66] Wilson LB Rojas DC Shatti S Tregellas JR . Greater neuronal responses during automatic semantic processing in schizophrenia. NeuroReport. (2013) 24:212–6. doi: 10.1097/WNR.0b013e32835eb688 PMC4086909 23399997

[67] Holmlund TB Cheng J Foltz PW Cohen AS Elvevåg B . Updating verbal fluency analysis for the 21st century: Applications for psychiatry. Psychiatry Res. (2019) 273:767–9. doi: 10.1016/j.psychres.2019.02.014 31207864

[68] Jackson RL Hoffman P Pobric G Lambon Ralph MA . The semantic network at work and rest: differential connectivity of anterior temporal lobe subregions. J Neurosci. (2016) 36:1490–501. doi: 10.1523/JNEUROSCI.2999-15.2016 PMC4737765 26843633

[69] Mkrtychian N Blagovechtchenski E Kurmakaeva D Gnedykh D Kostromina S Shtyrov Y . Concrete vs. Abstract semantics: from mental representations to functional brain mapping. Front Hum Neurosci. (2019) 13:267. doi: 10.3389/fnhum.2019.00267 31427938 PMC6687846

[70] Taylor JSH Rastle K Davis MH . Can cognitive models explain brain activation during word and pseudoword reading? A meta-analysis of 36 neuroimaging studies. Psychol Bull. (2013) 139:766–91. doi: 10.1037/a0030266 23046391

[71] Halliday M Hasan R . Language, context and text: Aspects of language in a social-semiotic perspective. Oxford: Oxford University Press (1989).

[72] De Beaugrande R Dressler W . Einführung in die Textlinguistik. Tübingen: Max Niemeyer (1981).

[73] Schrimpf M Blank IA Tuckute G Kauf C Hosseini EA Kanwisher N . The neural architecture of language: Integrative modeling converges on predictive processing. Proc Natl Acad Sci. (2021) 118:e2105646118. doi: 10.1073/pnas.2105646118 34737231 PMC8694052

